# Modeling lymphocyte homing and encounters in lymph nodes

**DOI:** 10.1186/1471-2105-10-387

**Published:** 2009-11-25

**Authors:** Valentina Baldazzi, Paola Paci, Massimo Bernaschi, Filippo Castiglione

**Affiliations:** 1 Istituto per le Applicazioni del Calcolo "M. Picone", Consiglio Nazionale delle Ricerche (CNR), c/o IASI-CNR, V.le Manzoni 30, 00185 - Rome, Italy; 2 Institut National de Recherche en Informatique et en Automatique (INRIA), Unité Rhône Alpes, 655 Avenue de I'Europe, Montbonnot, 38334 Saint Ismier Cedex, France

## Abstract

**Background:**

The efficiency of lymph nodes depends on tissue structure and organization, which allow the coordination of lymphocyte traffic. Despite their essential role, our understanding of lymph node specific mechanisms is still incomplete and currently a topic of intense research.

**Results:**

In this paper, we present a hybrid discrete/continuous model of the lymph node, accounting for differences in cell velocity and chemotactic response, influenced by the spatial compartmentalization of the lymph node and the regulation of cells migration, encounter, and antigen presentation during the inflammation process.

**Conclusion:**

Our model reproduces the correct timing of an immune response, including the observed time delay between duplication of T helper cells and duplication of B cells in response to antigen exposure. Furthermore, we investigate the consequences of the absence of dendritic cells at different times during infection, and the dependence of system dynamics on the regulation of lymphocyte exit from lymph nodes. In both cases, the model predicts the emergence of an impaired immune response, i.e., the response is significantly reduced in magnitude. Dendritic cell removal is also shown to delay the response time with respect to normal conditions.

## Background

Lymph nodes and Peyer's patches play key roles in the development of an appropriate and efficient immune response. Once an Antigen (Ag) is captured by Ag-processing cells, it is rapidly carried to the nearest lymph node, where it is presented to specific lymphocytes to trigger an immune response. The recognition phase must be highly efficient: within a few hours, it is necessary to find specific lymphocytes among a repertoire that includes a very large number of receptors [1,2]. The specific architecture of the lymph node and a fine-tuned balance between diffusion, chemotaxis, and receptor expression are the basis of this process.

Human lymph nodes are bean-shaped structures that range in size from a few millimeters to about 1-2 cm in their normal state. Internally, two main regions can be distinguished: the *medulla *and the *cortex*. The cortex can be further divided into an inner part, the *paracortex *(also called the *T cell area*), rich in T lymphocytes and an outer area, the *node cortex *that includes the *B cell area *consisting of follicles and germinal centers, where B cells are activated and differentiate [3]. T and B areas are identified by high concentrations of different chemokines (CCR7 and CXCR5, respectively) secreted by local stromal cells [1,4,5]. The whole structure is supported by a dense network of fibroblastic reticular cells that encloses small lymphatic channels of 10-15 *μ*m in diameter along which small molecules are thought to diffuse [6]. Macrophages, dendritic cells (DC), and some lymphocytes flow from the afferent lymphatic vessels, through the fibroblastic reticular cellular network, to the node cortex and the medulla, before leaving via the efferent lymphatic vessels. Most T and B cells, however, are too large in size and enter the lymph node mainly from the blood, through high endothelial venules (HEV) located inside the paracortex. During an infection, lymphocyte recruitment from the periphery is enhanced due to a widening of the primary arteriole feeding the lymph node [2]. Once inside the lymph node, B and T cells rapidly home in their own compartments, following a specific chemotactic gradient [1,4]. T helper cells (TH) are the fastest, with an average velocity of 11 *μ*m/min, followed by B cells with 6 *μ*m/min and DCs with a velocity of 3 *μ*m/min [7].

In the absence of an antigenic challenge, T and B cells randomly scan their respective areas for ~24-48 h before exiting the lymph node [8]. Entrance of antigens into the lymph node triggers a series of events leading to antigen recognition and the activation of an immune response.

Two distinct pathways for antigen delivery have been recognized. In general, an Ag-presenting cell picks up the antigen in peripheral tissues and migrates to the nearest lymph node in order to present the MHC-peptide complex to the T and B cells. In addition, antigens of low molecular weight (below 70 kDa) may enter the lymph node as soluble antigens and reach the T cell area directly through the fibroblastic reticular cellular meshwork, without any preliminary recognition. This delivery system is very efficient, and soluble antigens are detected inside the lymph node within a few minutes of infection. Here, specific resident DCs are able to capture the antigen well before Ag-presenting cells arrive from peripheral organs (on average, 8-12 h later) [9].

The immune response is initiated inside the T cell area, where antigens are first presented to the T cells, generally by DCs. After antigen recognition, peripheral dendritic cells undergo a change in the expression of their surface receptors. In particular, receptors for inflammatory chemokines are lost, and lymphoid receptors, especially the CCR7 receptor, are expressed [10]. As a consequence, Ag-presenting DCs are rapidly routed towards the T cell area, where the probability of meeting specific T cells is higher. In addition, once inside the lymph node, Ag-loaded DCs begin to release specific chemokines (e.g., MDC) to guide proximal active T cells [11,12].

B cell activation is initiated by an engagement of the B cell receptor either by a soluble or a membrane-associated antigen [13,14]. Following antigenic stimulation, B cells start to co-express the CCR7 receptor [15,16] and rapidly localize at the boundary between the T and B areas. Here, they can receive the right costimulation by T helper cells and start their proliferation and differentiation processes. Unstimulated lymphocytes rapidly pass through the lymph node to return to general circulation.

Lymphocyte egress from lymph nodes is still a subject of investigation. Recent studies suggest a key role of the Sphingosine-1-phosphate (S1P) molecule [17-19]. Its specific receptor S1P_1 _acts as a type of "pass filter" that selectively controls lymphocyte exit through efferent lymphatic vessels [19]. The receptor S1P_1 _is downregulated during lymphocyte activation [17], preventing Ag-specific lymphocytes from leaving the lymph node before an immune response has been mounted.

The variety of mechanisms and the large number of entities present in a lymph node make it difficult to understand the role played by each single component in the overall behavior. Models for several different aspects of lymphocyte motion have been developed [20-22]. Here, we present a model of the lymph node that is able to capture the interplay between different mechanisms, and which assures a fast and efficient immune response. We focus on lymphocyte recruitment and trafficking inside the lymph node, including specific cell diffusion properties, chemotaxis, and control of lymphocyte egress. We resort to a hybrid discrete/continuous approach that combines a stochastic agent-based description of cell interactions with a continuous model of molecular diffusion described by partial differential equations.

Agent-based models have a long history in immunology [23-25] because they have proven to be well-suited to handle complex nonlinearities and differences in individual cell characteristics. The choice of a hybrid approach is motivated by the need to explicitly consider the effects of chemotaxis on cells motility. This requires the introduction of a new timescale and, as a consequence, a distinct level of representation with respect to the inter-cellular interactions.

## Methods

The model currently includes the description of three types of cells: TH cells, B cells, and dendritic cells, plus four chemokines, CXCR5, CCR7, S1P, and MDC.

**Figure 1:**
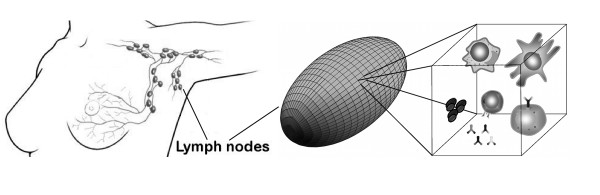
**Lymph node simulation mesh**. The three-dimensional ellipsoid lattice resembles the typical shape of a lymph node. The cube is a close-up of the volume unit where interactions between entities take place.

**Figure 2:**
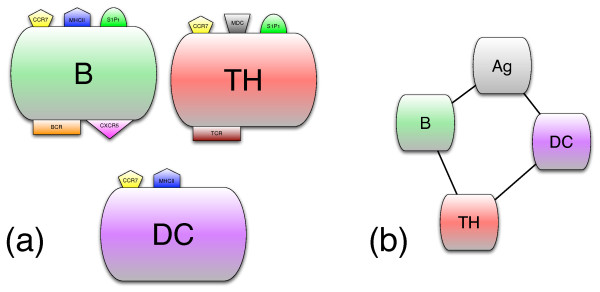
**Cells, their receptors, and the interactions among cells**. Panel (a) shows the cellular entities with their receptors: TCR and BCR are the antigen-receptors for the T and B lymphocytes, respectively; MDC, CCR7, and CXCR5 receptors are specific for the corresponding chemokines. S1P_1 _is the receptor for the molecule S1P that controls the exit of lymphocytes from the lymph node. In panel (b), connectors among the entities indicate that they interact.

Cells are represented as discrete agents with specific characteristics, that reside on a three-dimensional Cartesian mesh (Fig. 1). Each set of cells is endowed with receptors for antigen binding and one or more signaling receptors that allow them to "sense" the concentration of specific molecules. In addition, antigen-presenting cells, like B cells and DCs, have one MHC class II receptor for antigen processing (see Fig. 2). Receptors for antigen recognition and processing are represented by binary strings that are assigned randomly. On the other hand, the receptors for the signaling molecules are described by Boolean variables (e.g., receptor ON means able to sense the corresponding signal). Unlike antigen receptors, the expression levels of these receptors can change during simulation, depending on cell state.

Simple molecules like chemokines are represented by their spatial concentration that acts as a signal that triggers cellular interactions and movement. Unlike cells, molecules are not endowed with an internal description.

Each cell entity is always characterized by one of four possible states: 1) active, meaning that the cell can perform actions; 2) resting, meaning that the cell is waiting for the activation signal; 3) presenting, that is, an antigen-presenting cell exposes the antigen on the MHC class II molecule; 4) duplicating, meaning that the lymphocyte is in the mitotic cycle (see Table 1).

**Table 1:** Cellular internal states.

Entity	States			
	**Resting**	**Active**	**Presenting**	**Duplicating**

B		●	○	○

TH	●	○		○

DC		●	○	

The behavior of cells is determined by their internal state because each interaction condition is defined in terms of eligible states. Circles imply that they can occupy that state. A full circle indicates the initial state of the cell.

Cells change their internal state when they interact with other entities. Interactions are coded as probabilistic rules; each interaction requires the cells involved to be in a specific state and the interaction probability depends on the complementarity of their receptor strings, as measured by their Hamming distance. The interactions are local and many entities may sit on each single lattice site. In particular, for each lattice point, all interaction rules are executed in random order and a greedy paradigm is applied (i.e., cells that have interacted are taken out from the pool for the current time step). The list of all included interactions and signaling mechanisms is shown in Fig. 2.

The model description of chemotaxis is difficult due to the range of timescales involved in the phenomenon. Cells, in fact, are characterized by a diffusion coefficient much lower than for small signaling molecules [26], that can rapidly diffuse throughout the entire simulation volume.

The dynamics of molecules is considered deterministic, ruled by a simple heat-like partial differential equation, solved by an explicit finite difference scheme, describing a uniform diffusion process, with the addition of a degradation term,(1)

where *c *= *c*(*x*) is the concentration of chemokines (expressed in pM), *D *is the diffusion coefficient, and *λ *is proportional to the half-life of the molecules. We assume that *D *= 3000 *μ*m^2^/min and *λ *= 3 h [25,26]. Homing chemokines are continuously released by several sources that are randomly distributed inside the T and B areas. At each time step, a burst of new molecules is injected at each source point and immediately spread by diffusion over the simulation volume.

In contrast to chemokines, cells move individually at discrete time steps (one simulation time step ~30 mins real time). Chemotaxis is modeled in the following way. First, we compute the normalized difference of chemoattractant concentration between the current cell position and the neighboring lattice points, yielding a set of discrete percentages of total neighboring chemoattractant. The probability of moving in each direction is then partitioned according to the resulting percentages, and the direction of cell displacement is chosen stochastically. This results in cells moving with a higher frequency towards the maximum increase of chemoattractant concentration, and with a lower frequency away from chemoattractants, although this possibility is allowed. However, a minimal intensity for the chemotactic gradient is required in order to elicit a chemotactic response; a weak or null chemotactic field results in a simple random walk, similar to the one observed in two-photon microscopy imaging experiments [27,28]. The threshold is assumed to be cell-dependent. T cells are provided with a lower chemotactic sensitivity than B and DC cells (of a factor 10^3^) [1]. This allows T cells to move essentially randomly inside their area whereas B and dendritic cells are progressively guided towards the appropriate position (respectively, the B cell area and the T cell area) during the immune response [3,29].

Differences in cell mobility are also taken into account. Within the limit of our temporal resolution, the dynamics of cell-cell interactions is not relevant, and cell velocity is assumed to be constant during the entire simulation.

The transient effects of the chemotactic signals on a given cell are modeled by a modulation in the expression of the receptor signal, according to the cell's internal state. For instance, when a B cell presents the antigen, the expression of its CXCR5 receptor is turned off, and the CCR7 receptor is expressed in its place. In this way, B cells are redirected towards the boundary between the T and B cell areas, to receive the appropriate costimulation.

**Figure 3:**
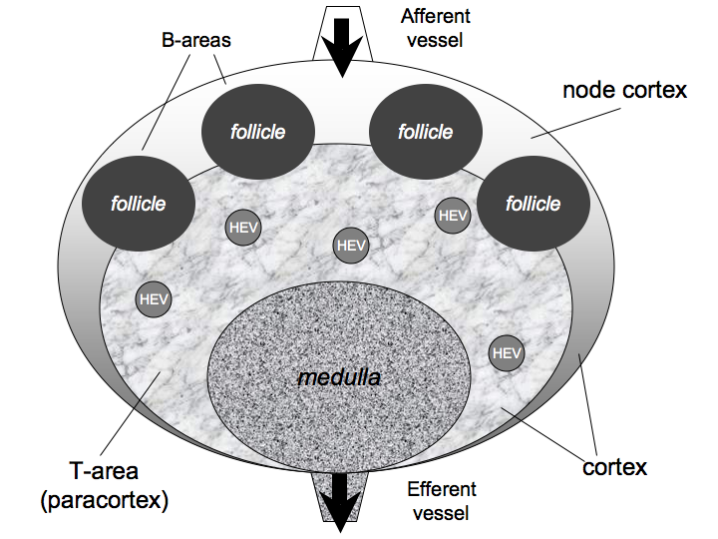
**Schematic representation of a lymph node internal architecture**. Schematic representation of the lymph node: T cell area (i.e., the paracortex) and a B cell area (i.e., the node cortex). A number of follicles are located in the node cortex, whereas the T cell area occupies the inner part. The medulla consists of a part of the T-area that allows cells to exit by the efferent vessel. T and B cells enter the lymph node mainly from the blood, through HEV located inside the paracortex (see text).

An analogous description applies to the control of lymphocyte egress from the lymph node, modeled by a change in the expression of the S1P receptor (i.e., the S1P_1_). Once a lymphocyte is found at an exit site, its S1P_1 _status is checked and, if it is expressed (i.e., the receptor is ON), the lymphocyte may leave the lymph node with a certain probability. When a TH or B cell succeeds in recognizing the Ag, the S1P receptor is deactivated thus preventing the cell from leaving the lymph node. Only once mature (i.e., after cloning), the lymphocyte's S1P receptor is turned on again, and Ag-specific lymphocytes can finally leave the lymph node. Exit control does not apply to DCs which simply exit at a constant low rate [17]. We use a three-dimensional mesh to model the typical ellipsoid shape of a lymph node (Fig. 1). The simulated volume corresponds to the typical dimensions of a small human lymph node (e.g., ~10 *μ*l). Internally, this is modeled as being composed of three regions: the T cell area (i.e., the paracortex), the B cell area, consisting of a number of follicles inside the node cortex, plus a region (i.e., the medulla) that contains the exit gates (randomly distributed) that lead to the efferent vessel (see Fig. 3). An afferent vessel enters the node cortex from the top of the simulation mesh.

The lattice unit is set equal to the average distance that a TH cell (i.e., the fastest cell entity in the model [7]) covers in one time step, i.e.,(2)

where *D*_*TH *_is the diffusion coefficient for the TH cell and Δ*t *is the time step.

At each time step, a constant number of naïve TH and B cells enter on the average the lymph node through several high endothelial venules, randomly distributed inside the T cell area [30]. Dendritic cells enter, instead, through the lymphatic flux, from the afferent vessel. A continuous renewal of lymphocytes and dendritic cells is provided by assuming a total population of about 10^6 ^cells (fluxes have been taken from [31]). To model the increase in lymphocyte recruitment during an infection, the incoming flux of TH and B cells is augmented by a factor that is proportional to the number of antigens inside the lymph node. We represent antigens as small immunogenic molecules. The model includes the description of both mechanisms for antigen delivery, based on a passive or active (i.e., DC-aided) transport mechanism. Soluble Ags are introduced into the lymph node immediately after infection, at random sites inside the T compartment. DC-presenting cells, instead, are inserted in the simulation volume shortly after the injection of soluble Ags, approximately 8-12 h later.

The parameters of the model can be classified into three categories: 1) parameters that correspond to the initial conditions of the system and that determine the problem under investigation; 2) parameters whose values are well-known and available from immunology literature; 3) unknown values (free parameters) which we set after a tuning procedure that starts with an initial guess based on empirical rules and iteratively improves by looking at the results of the simulations (see Table 2).

**Table 2:** Parameter set - parameters of the simulation.

PARAMETER	SYMBOL	MEANING	RANGE	VALUE (UNITS)	**REF**.
KNOWN VALUES					

DupStepB	*d*_B_	B's duplication steps	**N**	4	[34]
DupStepTH	*d*_TH_	TH's duplication steps	**N**	4	[34]
VelocityB	*v*_B_	B cells' velocity	**R**	6 *μ*m/min	[27,28]
VelocityTH	*v*_TH_	TH's velocity	**R**	11 *μ*m/min	[27,28]
VelocityDC	*v*_DC_	DCs' velocity	**R**	3 *μ*m/min	[25]
DiffCoeff	*D*	chemokines' diffusion coeff.	**R**	3·10^3 ^*μ*m^2^/min	[27,28]
HalfLife	*λ*	chemokines' half life	**R**	3 hrs	[25,26]
NInitial [B]	*B*(0)	B's initials per *μ*l	**N**	10^5 ^(cells/*μ*l)	[5,31]
NInitial [TH]	*TH*(0)	TH's initials per *μ*l	**N**	2·10^5 ^(cells/*μ*l)	[5,31]
NInitial [DC]	*DC*(0)	DC's initials per *μ*l	**N**	10^3 ^(cells/*μ*l)	[5,31]
NCELLS_B_ENTRY	*f*_B_	incoming flux of TH cells	**N**	31 cells/min	[5]
NCELLS_TH_ENTRY	*f*_TH_	incoming flux of TH cells	**N**	190 cells/min	[5]

ARBITRARY PARAMETERS					

NBITSTR	*l*	bit-string length	**N**	22 (*)	-
pDCAg	*p*_DC_	DC affinity to Ag (prob)	[0, 1]	0.04	-
nHEV	*n*_HEV_	numbers of HEV per *μ*l	**N**	4 (*μ*l^-1^)	[30]
pExit	*p*_Exit_	exit prob for cells	[0, 1]	0.44	-
ChemQty	*q*	released chemokines qty	**R**	10^8 ^mol (min src)^-1^	[35]

INITIAL CONDITIONS					

microL	*mL*	volume	**N**	10 (*μ*l)	-
deltaT	Δ*t*	time resolution	**R**	30 (min)	-
nSteps	*T*	time steps of the simulation	**N**	500 (steps)	-
AgInjFreq	*f*_Ag_	injection freq. of soluble Ags	**N**	4 (day^-1^)	-
AgInjQty	*n*_Ag_	soluble Ags per injection	**N**	100	-

Values that are taken from literature are provided with references. N and R indicate integer and real numbers respectively. (*) This choice of the bit string length represents a tradeoff between the computational cost of the simulation and the variability required for a realistic description of receptors repertoire.

The tuning of free parameters was performed by comparing the results against experimental values taken from the literature. For instance, i) an average lymphocyte residence time inside the lymph node of approximately 24-48 h [8]; ii) an outgoing flux per day of 25% of the population of cells [31]; (iii) an average homing time for newly-arrived B lymphocytes of about 10 hrs (estimated from [8]).

## Results and Discussion

Lymph node function is characterized as being in one of two distinct regimes or states, namely healthy or infected. Each state, and the transition between states, is characterized by specific timescales that result from the delicate combination of structural architecture, diffusion properties of components, and flux remodeling, as described above. Our model succeeds in reproducing the expected dynamics of the system with and without the presence of antigenic stimulation.

**Figure 4:**
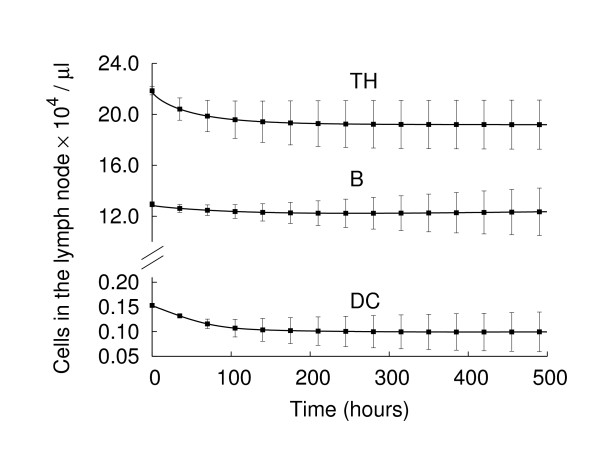
**Cell population dynamics in the absence of an antigenic stimulus**. In the absence of an antigen challenge, the system remains in a steady state. The corresponding cell density agrees with the expected values given the volume of our simulation space.

In the absence of an infection, lymphocytes continuously transit through lymph nodes during an endless surveillance of bodily tissues for possible infection. Circulation of the lymphocytes outside of the lymph nodes has been estimated to require nearly 30 min [10]. Lymphocytes spend approximately one day inside the lymph node before leaving again [8]. This continuous flux assures a constant renewal of the antigen expression repertoire and increases the probability of recognizing the antigens. In the absence of an infection, a balance between the incoming and the outgoing flux must be achieved in order to keep the cell population in a stable state. Fig. 4 shows the total (Ag-specific and unspecific) number of cells in the lymph node in the absence of antigenic challenge. Indeed, the population reaches a constant value, and the corresponding density agrees with the expected values, given the simulated volume i.e., ~2·10^5 ^TH cells, 10^5 ^B cells and 10^3 ^DCs [5,31]. In particular, on average, 25-30% of TH cells leave the lymph node in one day, whereas about 10% of B cells are refreshed.

**Figure 5:**
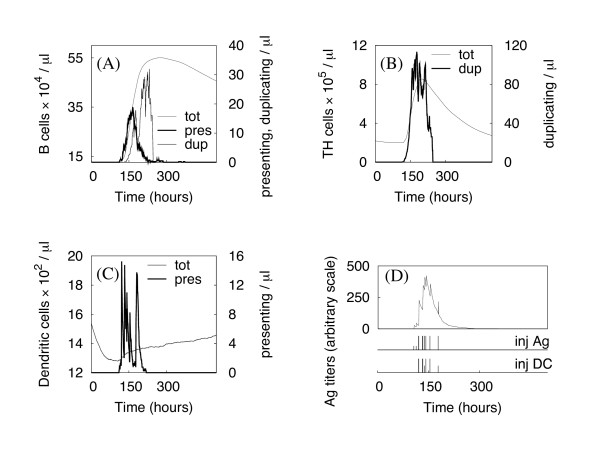
**Simulation of an immune response during an infection**. The different panels show the dynamics of B cells, TH cells, and DCs (panels A, B, C, respectively), in the different states. *Tot *stands for total cell number, *pres *are the cells currently presenting the antigen at their surface, whereas the *dup *cells are the duplicating ones. Panel D shows the Ag concentration inside the lymph node, identifying the time window of infection. Vertical lines show the times of Ag and DC injection (inj Ag and inj DC, respectively).

**Figure 6:**
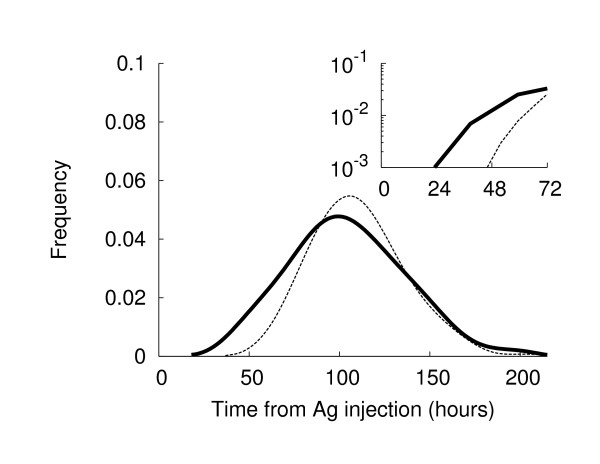
**Cell Duplication frequency during an immune response**. TH cells (continuous line) start cloning 24-30 hrs after Ag injection, whereas the B cell response (dashed line) is delayed by roughly one day, as reported in the literature. Results are obtained from 500 simulations.

The arrival of Ags breaks the equilibrium, triggering a set of well-balanced processes aimed at speeding antigen presentation and immune system activation. Fig. 5 shows the dynamics of the various cells populations in the presence of antigenic stimulus. Vertical bars in panel (D) show the injection times for soluble antigens or DCs loaded with antigen peptides. As soon as the first antigens reach the lymph node, the lymphocyte density begins to increase due to the strengthened incoming flux. Most antigens are captured by DCs and B cells, as can be seen from the sudden rise in the number of presenting cells. The immune response initiates when antigen-specific lymphocytes start to duplicate. Fig. 6 shows the probability distribution, obtained from 500 simulations, of TH and B cell duplication as a function of time since Ag injection. TH duplication is shown to initiate almost one day after Ag injection, as reported by [5,32], whereas B cell response, requiring costimulation, appears delayed. A shift of about 24 h (see inset plot of the same figure) is evident between the B and TH responses, in reasonable agreement with the experimental data that observed B cell duplication ~ two days after infection [3,5].

As long as new antigens are detected, the immune response persists: the total lymphocyte number increases monotonically up to 5 times the original value [2,8], and cells continue to duplicate, increasing the pool of available Ag-specific cells (see Fig. 5). Once the infection is over, the immune system and the lymph node recover the original healthy state. After about 200 h, in the presented simulation, Ag concentration has nearly vanished, and the incoming cell flux begins a decrease to its original value. The total number of TH and B cells gradually reduces and recovers its normal value within a couple of weeks. B cells leave the lymph node at a lower rate because of their reduced motility with respect to TH cells.

### In silico experiments

We now use the model to investigate different scenarios by perturbing the normal function of the system and looking at the emergent response. Several experiments can be designed in response to specific questions. In the following, we propose two distinct experiments aimed at investigating the biological role of DC and S1P_1_-control of lymphocyte egress on the onset of an efficient immune response.

#### The role of dendritic cells

Dendritic cells are recognized as the most efficient antigen-presenting cells. Their function is of primary importance for the activation of specific lymphocytes inside a lymph node. In the following, we show the effect of removing all DCs from the lymph node at a given time. Depending on the delay from Ag injection, the effect on the immune response varies.

**Figure 7:**
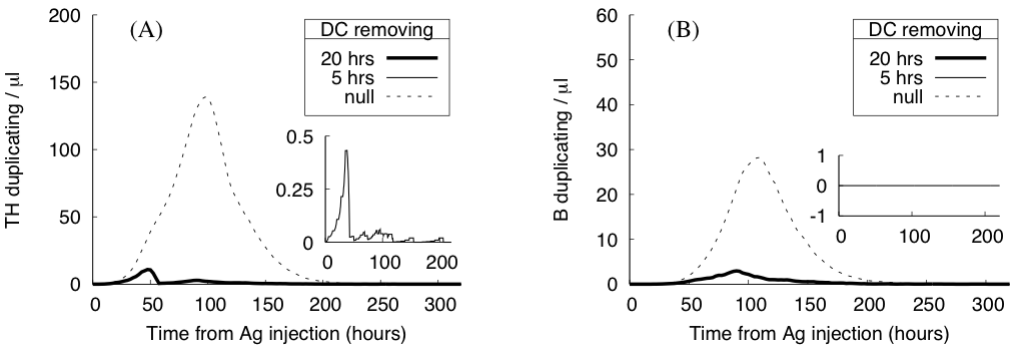
**In silico experiment: the role of DC cells**. Number of Ag-specific duplicating lymphocytes after DC removal performed 5 (inset plot) or 20 hrs after Ag injection. Results show the average number of duplicating TH (panel A) and B (panel B) cells. If DCs are removed too early, the immune response fails to mount.

Simulation results are showed in Fig. 7. When the DCs are removed late in the simulation time, e.g., 20 h after Ag injection, the immune system still has time to mount a response. Both TH and B cell duplication are observable, even if they appear reduced and slightly delayed with respect to the unperturbed situation (see the dashed line for comparison). If DCs are instead removed too early, the immune response is completely shut off, because DCs do not have enough time to activate TH cells. Only when active, TH cells are able to stimulate B cells and to eventually duplicate, provided that the right signal is given. Moreover, in an early DC removal, the few active THs are unlikely to meet the specific B cells that fail to clone in the absence of the essential costimulation. Therefore, in this situation, the model predicts TH cell activation as a pivotal step in the onset of a correct immune response.

#### Lymphocyte exit control: role of the S1P receptor

The mechanisms that regulate lymphocyte exit from the lymph node have been subject to intense research activity in the last few years. Several studies implicate the Sphingosine-1-phosphate receptor (S1P_1_) as a crucial element for the regulation of the exit mechanism of lymphocytes from the lymph node and highlight its importance in the selective retention of Ag-specific lymphocytes during an infection.

**Figure 8:**
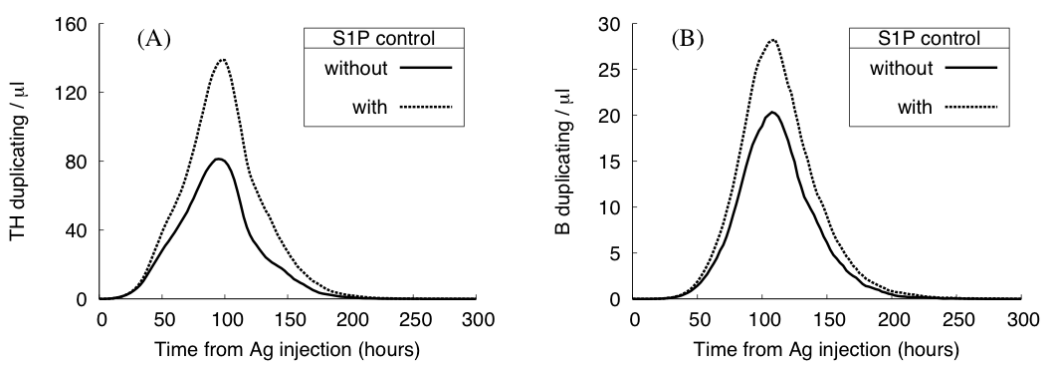
**In silico experiment: the role of lymphocyte exit control**. Number of Ag-specific duplicating lymphocytes with (dashed lines) and without (solid lines) S1P_1 _exit control. Results show the average number of duplicating TH (panel A) and B (panel B) cells. Blocking S1P_1 _downexpression, the magnitude of the immune response is greatly reduced.

To investigate the impact on the overall adaptive immune response, we block the S1P_1 _downexpression during TH and B cell activation. As a result, all lymphocytes may leave the lymph node once they reach an efferent vessel, regardless of their antigen specificity. Fig. 8 compares the average number of duplicating TH and B cells during an immune response in the presence and absence of the S1P_1 _based mechanism described above. By blocking S1P_1 _downexpression, the magnitude of the immune response is greatly reduced and the number of responding TH cells is nearly halved. As a consequence, the number of antibodies produced also decreases, thus weakening the organism's protection against antigen infection.

However, the S1P_1 _control does not seem to affect the onset and overall dynamics of the immune response: the timing of lymphocyte encounters appears to be mainly determined by the geometry and the diffusion properties of the system rather than the Ag-specificity of its cell population.

## Conclusion

We have developed a model that reproduces some aspects of the immune response and the behavior of cell/antigen motility within a lymph node. In particular, we focus on the mechanisms that determine the onset of a primary immune response, from Ag delivery to B cell activation and duplication and the observed lymph node shrinking after an immune response. We stress that the obtained T and B cell responses at the correct times are emergent properties of a quasi-realistic description of lymphocyte density, interactions, and motion (including chemotaxis and diffusion characteristics), combined with a schematic description of lymph node compartmentalization. This is a key difference from previous work [5] in which the correct timing of the immune response was somehow hard-coded in the simulator.

The model provides interesting insights into the role played by DCs and by the regulation of lymphocyte exit from lymph nodes on the resulting immune response. We show an impaired immune response when one of these mechanisms is perturbed. DC removal at early times produces large effects, with an immune response that is greatly delayed and reduced in magnitude due to the lack of active TH cells that are able to provide the right costimulation of B lymphocytes. S1P_1 _control of specific cells, instead, affects essentially the magnitude of the immune response rather than the timing, by decreasing the overall number of specific lymphocytes that can participate in Ag detection. In the same spirit, many other experiments could be planned to investigate, for example, the role of chemotaxis and transient receptor expression on the different phases of Ag presentation.

The current model represents a first attempt to comprehensively sketch fundamental aspects of lymph node function based on a few cellular component and molecular mechanisms. Only essential pathways have been included in the current version of the model, and further work is needed to enhance the description of the rich variety of behaviors that can be observed in a real immune response. Future improvements include a better description of B cell activation (by macrophages or dendritic cells) and differentiation into plasma and memory cells. In particular, it would be interesting to examine the impact of T-independent B activation mechanisms on the emergence of a humoral immune response [33]. Moreover, the presence of memory lymphocytes has important consequences for the dynamics of a second immunization. With the inclusion of memory cell generation in the model, differences in lymphocyte population and Ag-presentation efficiency between the primary and secondary responses could be analyzed. Specific Ag features (e.g., if it is a bacterium or a virus) and other related biological processes could also be taken into account to address specific questions. In parallel with progress in lymph node understanding, this simulator can be a useful tool to test new hypotheses, investigating the effect of additional mechanisms on the resulting immune response.

## Availability and requirements

- Name: homing

- Home page: http://www.iac.cnr.it/~filippo/lymphnode.html

- Operating system(s): Unix/Linux, Windows, Mac OS

- Programming language: ANSI C

## Authors' contributions

VB, FC, and MB conceived and designed the project; VB developed the code; VB, PP and FC performed the simulations and analyzed data; all authors wrote the paper.
